# Acute intrathoracic gastric volvulus with retrograde gastric intussusception: A case report of a rare surgical emergency with review of the literature

**DOI:** 10.1016/j.ijscr.2020.06.042

**Published:** 2020-06-12

**Authors:** Giovambattista Caruso, Sebastiano Caramma, Angelo Zappalà, Domenico Zerbo, Giuseppe Evola, Carlo Reina, Giuseppe Angelo Reina

**Affiliations:** a General Surgery Department, Santissimo Salvatore Hospital (ASP Catania), Paternò, Catania, Italy; b General and Emergency Surgery Department, Garibaldi Hospital, Catania, Italy

**Keywords:** Gastric volvulus, Upside-down stomach, Gastrogastric intussusception, Emergent surgery, Subtotal gastrectomy, Case report

## Abstract

**Introduction:**

The gastric volvulus is a rare condition in which the stomach, or part of it, rotates on its axis, for over 180°, constituting a surgical emergency. Even more rare is gastro-gastric intussusception. A delay in their diagnosis and treatment can have fatal consequences

**Presentation of case:**

An 82-year-old woman was admitted to the Surgery Unit with a two-day history of abdominal pain associated at first with coffee vomiting and, subsequently, with unproductive retching and oligoanuria. Physical examination showed severe dehydration, fever, at the abdominal level, palpation caused a marked tenderness of all quadrants, with signs of peritonism.

Laboratory test showed showed neutrophilic hyperleukocytosis and high C reactive protein level. Abdominal computed tomography revealed an acute intrathoracic gastric volvulus and a gastrogastric intussuception. The patient was submitted to exploratory laparotomy, subtotal gastrectomy with Roux en Y anastomosis and simple plastic of the esophageal hiatus. At the end of the surgery, however, the patient died of your septic shock.

**Discussion:**

The traditional treatment for a patient with acute gastric volvulus is an immediate surgical intervention to derotate the stomach and prevent vascular insufficiency.

In the presence of necrosis or gastric perforation, resection should be performed.

The few cases of gastrogastric intussusception described in the literature have been treated with sub-total gastrectomy and gastro-jejunal anastomosis. Any delay in diagnosis and treatment can prove fatal.

**Conclusion:**

Intrathoracic Gastric Volvulus and, even more, retrograde gastrointestinal intussusception are very rare pathologies, difficult to diagnose.

## Highlights

• The acute intrathoracic gastric volvulus is a rare condition.
• The gastrogastric intussusception is very very rare.
• The gastric volvulus and the gastrogastric intussusception are surgical emergencies.
• Any delay in diagnosis and treatment can prove fatal.
• Emergent laparotomy or laparoscopy is considered the correct treatment.

## 1 Introduction

The gastric volvulus, described first by Berti in 1886, is a rare condition in which the stomach, or a part of it, rotates on its axis, for over 180°, constituting a surgical emergency due to the risk of ischemic necrosis, perforation and severe sepsis, with a mortality ranging from 30% to 50% [[1], [2], [3], [4],^20^].

A gastro-gastric intussusception is more rare.

In literature, only 11 cases are reported from 1950 to 2019 [5].

A delay in their diagnosis and treatment can have fatal consequences.

The present work has been reported in accordance with the Surgical Case Reports (SCARE) criteria [6].

## 2 Presentation of case

In November 2019, a 82-year-old woman, without comorbidity, was admitted to the Emergency Department, for violent epigastric pain, which had arisen about 24 h before, after the meal, associated at first with coffee vomiting and, subsequently, with unproductive retching and oligoanuria.

For some years now, she reported lack of appetite, postprandial abdominal bloating, difficulty erupting and, sporadically, intermittent epigastric pain that she treated with self-medicated drugs.

Physical examination showed a severe dehydration. Temperature: 37.8 °C. BP: 90/50 mmHG. HR: 140 bpm. GCS: 8. Marked tenderness at palpation of the all abdominal quadrants, with signs of peritonism. Anuric patient.

Blood tests showed neutrophilic hyperleukocytosis (WBC: 30,000/mmc), an increase in creatinine (1.47 mg/dl), CRP (139.7 mg/l) and LDH (504 IU/l) values.

CT of the thorax and abdomen with contrast showed a voluminous sliding hiatal hernia with herniation of nearly the entire stomach in the retrocardiac seat. A possible diagnosis of acute gastric volvulus was made. The presence of retrograde gastro-gastric invagination at the level of the antrum was also reported.

**Fig. 1:**
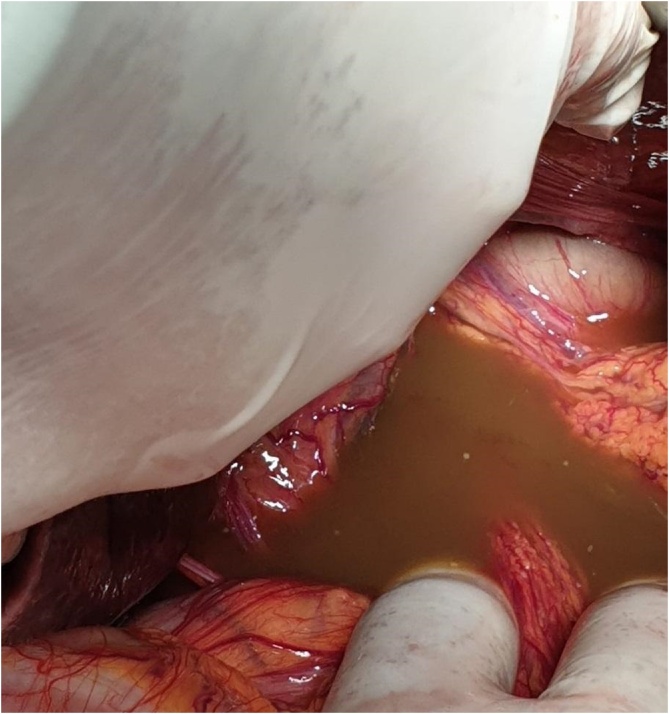
Intraoperative view.

**Fig. 2:**
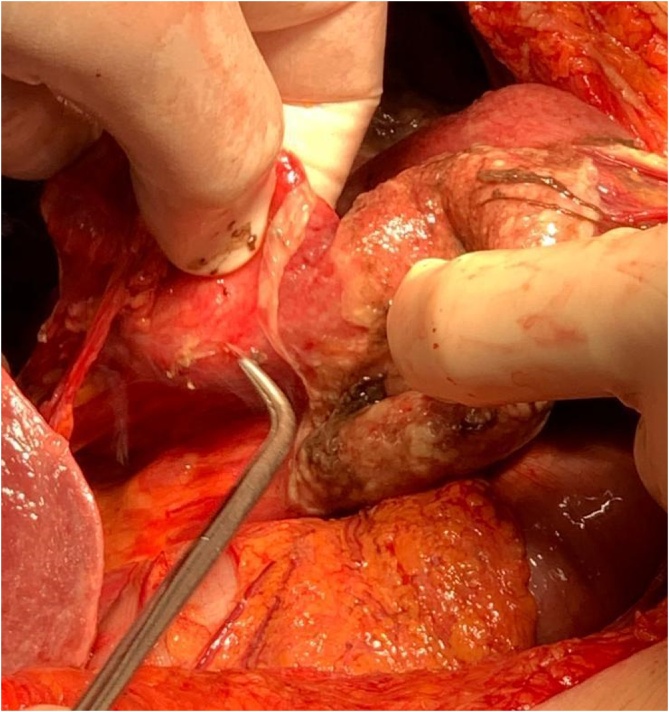
Intraoperative view.

**Fig. 3:**
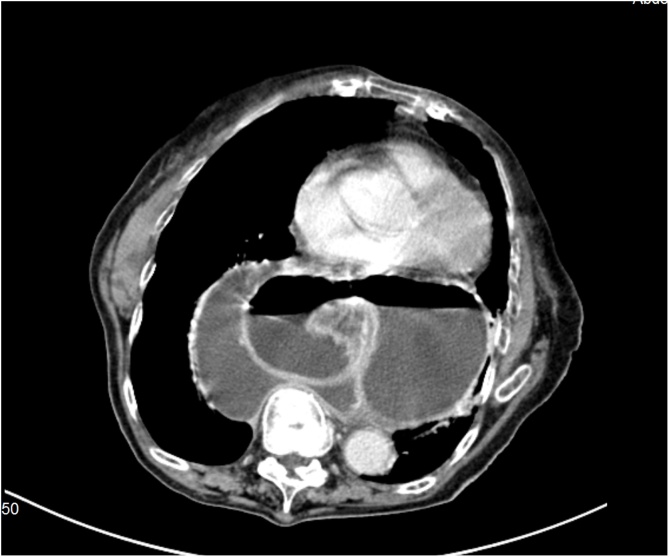
Herniation of nearly the entire stomach in the retrocardiac seat. Axial plane.

**Fig. 4:**
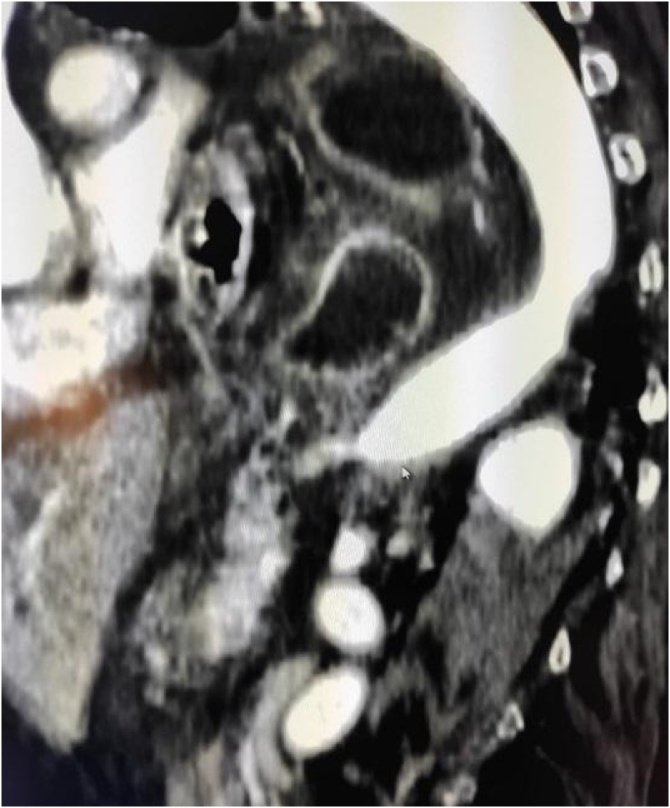
Herniation of nearly the entire stomach in the retrocardiac seat. Longitudinal plane.

**Fig. 5:**
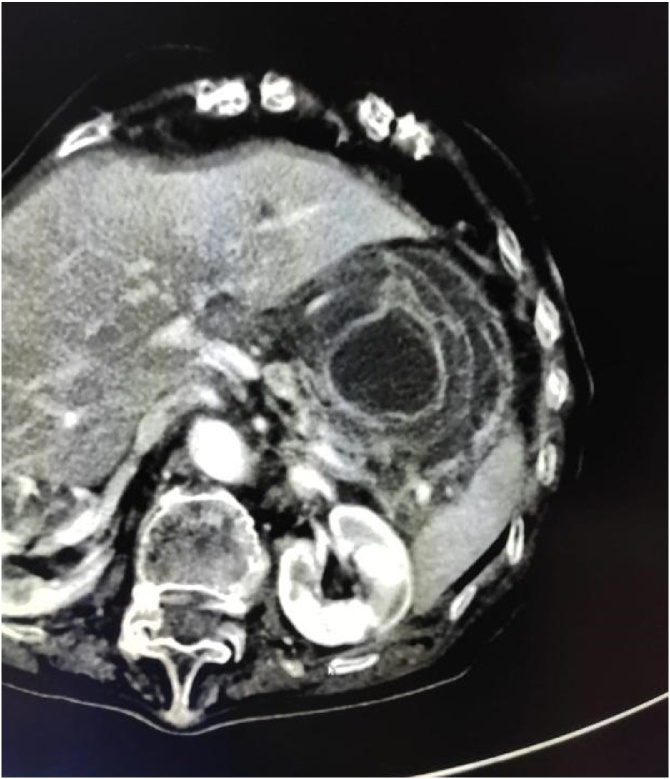
The target-shaped image of the gastrogastric intussusception.

Considering severe sepsis and rapid declin of the general conditions, after rapid resuscitation with infusion of liquids, electrolytes and broad-spectrum antibiotic therapy, two hours after admission to the emergency department the patient was subjected to exploratory laparotomy which revealed an abundant intra-abdominal bilious effusion and voluminous hiatal hernia with complete herniation in the thorax of the stomach, twisted on its long axis and with ischemic necrosis of the antrum (Fig. 1, Fig. 2, Fig. 3, Fig. 4, Fig. 5.

The gastric antrum appeared invaginated in the gastric body and with punctiform perforation of its anterior wall. A large amount of enteric fluid in the posterior mediastinum, with mediastinitis, was also found at the reduction of the hernia in the abdomen. After abdominal washing and a subtotal gastrectomy with Roux en Y anastomosis, a simple plastic of the esophageal hiatus and positioning of abdominal drainages were performed. The operative time was 120 min.

At the end of the surgery, however, the patient died of septic shock.

## 3 Discussion

Gastric volvulus is a rare, life-threatening clinical event, due to the risk of severe complications.

The incidence is higher since the fifth decade of life, although 10–20% of cases occur among children under the age of 1 year [21].

Volvulus can be classified as **idiopathic** (10–30% of cases), linked to the laxity of the perigastric ligaments, and **secondary** (more common) to gastric or diaphragmatic anomalies.

Conventionally, gastric volvulus is imagined as an intra-abdominal condition. However, although uncommon, an intrathoracic variant is observed and is linked to a herniation of the stomach in the chest through a diaphragmatic defect (hiatal hernia in most cases) [3].

The Upside-down stomach (UDS) is the rarest form of hiatal hernia (<5%), characterized by the herniation of the entire stomach, or most of it, in the posterior mediastinum and would predispose to intrathoracic gastric volvulus [20].

As well as hiatal hernias, UDS can give a wide variety of symptoms such as retrosternal pain, heartburn, post-prandial fullness, dysphagia, nausea, vomiting, anemia and mass-effect symptoms [22,23].

According to Singleton, gastric volvulus can be classified as **organoaxial** (59%), when the stomach rotates around the pylorus and the gastroesophageal junction and **mesenteroaxial** (29%), when the stomach rotates along the longitudinal line parallel to the small gastric curve, or mixed (12%) [1,19,21].

The gastric volvulus, clinically, can present itself as an acute abdomen or as a chronic intermittent recurrent pathology.

Acute gastric volvulus presents itself with a sudden and violent pain in the upper left quadrant or at the base of the left hemithorax. Other symptoms may be wheezing, unproductive retching, dehydration and prerenal insufficiency [21,22].

The most common complications of acute gastric volvulus are incarceration, strangulation and perforation.

The mortality rate varies between 30% and 50% and it increases to 60% if strangulation and ischemia occur [21].

Because of its rarity, a doctor with no deep experience, could relate this pathology to other non-surgical abdominal diseases or even to an acute coronary syndrome.

Hence an accurate anamnesis, a detailed clinical examination and a careful interpretation of the radiological images are important to face a patient who has vomit and high abdominal pain [4].

In 1904, Borchardt described the triad of acute epigastric pain, unproductive retching and the difficulty or impossibility of positioning a nasogastric tube.

This triad is found in 70% of patients with acute organoaxial volvulus.

Chest x-ray highlights the presence of abdominal viscera that have risen in the chest [3,23].

Other tests, often not performed in acute, are the barium contrast studium and digestive endoscopy [4].

The Chest and Abdomen CT allows to have an immediate diagnosis, to know the extent of the herniation, to put the right surgical indication, facilitating so preoperative planning [1,3,4,23].

The traditional treatment is an immediate surgical intervention to derotate the stomach and to prevent vascular insufficiency.

In the presence of necrosis or gastric perforation, resection should be performed.

At the same time, a reduction of the hernia and repair of the diaphragmatic defect should be made.

The stomach is then fixed to the anterior abdominal wall by simple suturing or by placing a gastrostomy tube.

Open or laparoscopic surgery can be superimposed in terms of results. However, in urgency, the open treatment is often preferred.

In elderly patients and high surgical risk ones, an attempt at medical management may be helpful.

The simple positioning of a nasogastric tube or, an endoscopic decompression with PEG positioning can be decisive [1].

A much rarer clinical condition is gastrogastric intussusception.

In adults, it only occurs 5% of cases and less than 10% of these cases affect the gastro-duodenal region [7].

Patients with this condition often have nonspecific symptoms, typically characterized by epigastric pain and vomiting [5].

If untreated, intussusception can cause ischemia of the invaginated bowel wall and consequent perforation with peritonitis.

The typical tomographic sign of intussusception is the target-shaped image [6].

The evidence from previous studies also indicates that a soft tissue growth, malignant or benign, is a typical concurrent finding and lead point. However, cases of gastrogastric intussusception with alternative pathophysiological mechanisms have been reported [5].

The presence of hiatal hernia, the laxity of the gastric ligaments, the increased intra-abdominal pressure and previous diaphragmatic surgery are considered predisposing factors [[7], [8], [9]].

In 2017, Behrooz, described the first case of gastrogastric intussusception from vascular congestion, assuring that portal hypertension, ascites, and the presence of a hiatal hernia triggered the invagination in the absence of an underlying neoplasm [5].

Hiatal hernia, in the absence of a tumor that acts as an invaginating head, is a well-known risk factor of esophagogastric intussusceptions as reported by Ghahremani and El-Hajj [10] (Table 1).

**Table 1:** Gastrogastric intussusception reported in literature from 1950.

Author	Age/Sex	Presentation	Diagnosis	Radiological images	Histological findings
Thompson [11]	72 M	Epigastric pain, nausea, vomiting.	Laparotomy	Not stated	Peduncolated intragastric tumor
Raw [12]	66 F	Epigastric discomfort, vomiting.	Laparotomy	Not stated	Malignant gastric papilloma
Grundy [13]	78 F	Weight loss, Dysphagia, vomiting, epigastric pain.	Fluoroscopy	Fundal mass intussusception into antrumwith pseudopedicle	Leyomioma
	76 F				
Javors [14]	81 F	Anaemia	Single contrast UGI series	Foreshortening of stomach with pseudopedicle, antral ovoid mass, coiled spring appearance	Leiomyoma with leiomyoblastomatous elements
Vikram [15]	65 F	Epigastric pain, nausea, vomiting, epigastric mass	Double contrast barium meal, CT abdomen	Bird’s beak appearance, invagination of wall of greater curve into gastric lumen	Gastrointestinal stromal tumour
Shanbhogue [16]	83 F	Melaena, weight loss, anaemia	CT abdomen	Target sign	Gastric carcinoma
Eom [17]	73 F	Vomiting, General weakness, sepsis	OGD, CT abdomen	Polypoid mass with a vascular pedicle	Gastric adenocarcinoma
Jo [18]	82 F	Chest pain, vomiting	CT abdomen	Mass in body of stomach telescoping into antrum	Primary gastric Lymphoma
Davila [7]	77 F	Fever, abdominal discomfort, left lateral abdominal mass	MR abdomen	Target sign	Tubulo-villous adenoma
Behrooz [5]	68 M	Abdominal pain, disfagia, vomiting, general weakness	CT abdomen	Filling defect with vascular pseudopedicle image	Vascular congestion of the gastric wall

This pathophysiological mechanism is what we postulate may have occurred in the patient of the clinical case reported by us, i.e. the vascular congestion of the gastric wall, linked to the gastric volvulus, in association with the hiatal hernia and the laxity of the ligaments, behaved as a trigger intussusception.

The treatment of adult invaginations is generally surgical and, given the high incidence of underlying malignancies, consists in the resection of the intestinal segment involved and anastomosis. The few cases of gastrogastric intussusception described in the literature, have been treated with sub-total gastrectomy and gastro-jejunal anastomosis.

In the reported case, the patient had an intrathoracic organoaxial gastric volvulus associated with retrograde gastrogatric intussusception with ischemic necrosis and perforation at the level of the antrum. The chronicity and the intermittence of the symptomatology have never prompted the patient to undergo a previous diagnostic investigations. Only in the face of the presence of violent epigastric and thoracic pain, unproductive retching, dehydration and decay of the general status, she resorted, with considerable delay, to medical treatment, but the gastric volvulus and invagination, which occurred in the hours prior to hospitalization, contributed to the necrosis and the consequent perforation with peritonitis, mediastinitis and a septic shock resulting fatal for her. Hence the importance, in the face of a patient with violent abdominal pain, with unproductive vomiting, not to neglect the diagnostic hypothesis of an acute gastric volvulus, because any delay in diagnosis and treatment can prove fatal.

## 4 Conclusions

Intrathoracic Gastric Volvulus and, even more, retrograde gastrointestinal intussusception are very rare pathologies. A delay in their diagnosis and treatment can have fatal consequences such as gastric ischemia and perforation. In consideration of these severe complications, we underline the importance of a correct diagnostic framework with a detailed anamnesis, a meticulous physical examination and a careful analysis of the radiological tests when you are faced with a patient who presents unproductive vomiting and sudden epigastric pain.

## Declaration of Competing Interest

All the authors certify that there is no conflict of interest regarding the material discussed in the manuscript.

## Funding

All the authors declare that this research didn’t receive any specific grant from funding agencies in the public, commercial, or not-for-profit sectors.

## Ethical approval

Ethical approval has been exempted by our institution because this is a case report and no new studies or new techniques were carried out.

## Consent

Written informed consent was obtained from the patient for publication of this case report and accompanying images.

## Author contribution

Giovambattista Caruso: Operated on the patient, drafting the manuscript.

Sebastiano Caramma: Operated on patient.

Domenico Zerbo: Literature search.

Angelo Zappalà: Literature search and revising the manuscript.

Giuseppe Evola: Revising the manuscript.

Carlo Reina: Drafting the manuscript.

Giuseppe Angelo Reina: Clinical supervision and consultation.

## Registration of research studies

This case report does not require registration as a research study.

## Guarantor

The guarantor for this case report is Giovambattista Caruso.

## Provenance and peer review

Not commissioned, externally peer-reviewed.
